# Reply to: Association of accelerometer-measured physical activity intensity, sedentary time, and exercise time with incident Parkinson’s disease: need more evidence

**DOI:** 10.1038/s41746-024-01155-z

**Published:** 2024-06-18

**Authors:** Mengyi Liu, Xianhui Qin

**Affiliations:** Division of Nephrology, Nanfang Hospital, Southern Medical University; National Clinical Research Center for Kidney Disease; State Key Laboratory of Organ Failure Research; Guangdong Provincial Institute of Nephrology, Guangdong Provincial Key Laboratory of Renal Failure Research, Guangzhou, 510515 China

**Keywords:** Parkinson's disease, Risk factors

We thank Chen et al. for their attention and thoughtful comments on our study^1^. We wholeheartedly agree with Chen et al. that more evidence is needed to support the relationship of physical activity and sedentary time with incident Parkinson’s disease (PD). Our response to these comments is as follows.

Firstly, while sleep disturbances seem to be closely related to PD in observational studies^2,3^, there is no convincing evidence of any causal relationship of self-reported and objective sleep traits with the risk of PD in Mendelian randomization analysis^4,5^. However, genetic variants in Mendelian randomization analysis may not be an accurate substitute for sleep because genes can only account for a relatively small proportion of sleep phenotypic variation, and thus, the non-significant relationship may be attributed to insufficient statistical power. As such, the association between sleep and PD risk remains uncertain. Of note, only 2415 (2.5%) participants in our study^1^ were diagnosed with sleep disorders (as ascertained by primary care data, hospital inpatient data, death register records, or self-reported medical conditions based on ICD code G47) prior to the time of accelerometer measurement, and 10 of these participants developed incident PD during follow-up. As a result, we were unable to provide further evidence on the relationship between accelerometer-measured light physical activity (LPA), moderate-to-vigorous physical activity (MVPA), and sedentary time with incident PD in subgroups with sleep disorders, which may limit the generalizability of the findings to people with severe sleep disorders. Nevertheless, the independent association of LPA, MVPA, and sedentary time with the risk of incident PD did not substantially change by further adjusting for the self-reported five sleep behaviors^6^, including sleep duration, chronotype preference, insomnia, snoring, and daytime sleepiness. In addition, the association of LPA, MVPA, and sedentary time with incident PD was consistent across participants with 0 to 5 healthy sleep factors (all *P* for interaction >0.05)^6^. These results suggest that the possibility that our findings were materially influenced by sleep should be small.

Secondly, the independent association of LPA, MVPA, and sedentary time with the risk of incident PD did not change substantially by further adjusting for coffee and tea consumption collected via standardized and validated touchscreen Food-Frequency Questionnaire at baseline. Moreover, in the further stratified analyses, coffee intake (consumers *vs*. non-consumers) and tea intake consumers vs. non-consumers did not significantly modify the association of LPA, MVPA, and sedentary time with incident PD (all *P* for interaction >0.05). That is to say, tea and coffee intake did not significantly affect the association of LPA, MVPA, and sedentary time with incident PD.

Finally, we acknowledged that although our study has comprehensively adjusted for important confounding factors, it cannot exclude the possibility of potential residual confounding of other unrecorded but important covariates.

Overall, our study shows that higher accelerometer-measured MVPA and LPA were associated with a lower risk of PD, while higher sedentary time was associated with a higher risk of PD. We agree with Chen et al. that future studies considering more potential confounding factors are needed to confirm our finding.

## Reporting summary

Further information on research design is available in the Nature Research Reporting Summary linked to this article.

### Supplementary information

Reporting Summary

## Data Availability

The UK Biobank data are available on application to the UK Biobank.
